# Intra‐ and intervariability in beam data commissioning among water phantom scanning systems

**DOI:** 10.1120/jacmp.v15i4.4850

**Published:** 2014-07-08

**Authors:** Yuichi Akino, John P. Gibbons, Daniel W. Neck, Connel Chu, Indra J. Das

**Affiliations:** ^1^ Department of Radiation Oncology Indiana University School of Medicine Indianapolis IN; ^2^ Department of Medical Physics Mary Bird Perkins Cancer Center Baton Rouge LA USA

**Keywords:** quality assurance, scanning water phantom, beam data commissioning

## Abstract

Accurate beam data acquisition during commissioning is essential for modeling the treatment planning system and dose calculation in radiotherapy. Although currently several commercial scanning systems are available, there is no report that compared the differences among the systems because most institutions do not acquire several scanning systems due to the high cost, storage space, and infrequent usage. In this report, we demonstrate the intra‐ and intervariability of beam profiles measured with four commercial scanning systems. During a recent educational and training workshop, four different vendors of beam scanning water phantoms were invited to demonstrate the operation and data collection of their systems. Systems were set up utilizing vendor‐recommended protocols and were operated with a senior physicist, who was assigned as an instructor along with vendor. During the training sessions, each group was asked to measure beam parameters, and the intravariability in percent depth dose (PDD). At the end of the day, the profile of one linear accelerator was measured with each system to evaluate intervariability. Relatively very small (SD < 0.12%) intervariability in PDD was observed among four systems at a region deeper than peak (1.5 cm). All systems showed almost identical profiles. At the area within 80% of radiation field, the average, and maximum differences were within ± 0.35% and 0.80%, respectively, compared to arbitrarily chosen IBA system as reference. In the penumbrae region, the distance to agreement (DTA) of the region where dose difference exceed ± 1% was less than 1 mm. Repeated PDD measurement showed small intravariability with SD < 0.5%, although large SD was observed in the buildup region. All four water phantom scanning systems demonstrated adequate accuracy for beam data collection (i.e., within 1% of dose difference or 1 mm of DTA among each other). It is concluded that every system is capable of acquiring accurate beam. Thus the selection of a water scanning system should be based on institutional comfort, personal preference of software and hardware, and financial consideration.

PACS number: 87.53.Bn

## I. INTRODUCTION

In recent decades, radiotherapy technologies and dose calculation algorithms have been significantly improved. Accurate commissioning of radiotherapy beam parameters^(1)^ and treatment planning systems (TPS) has become important for dose calculation needed for patient treatment. Especially for special technologies such as intensity‐modulated radiotherapy (IMRT),^(2)^ volumetric‐modulated arc therapy (VMAT),^(3)^ and stereotactic radiotherapy (SRT),^(4)^ and also for flattening filter‐free (FFF) beams,^(5)^ accurate beam data commissioning for modeling is essential. Although there have been improvements in standardizing linear accelerator output characteristics, machines may not have identical beam characteristics, even for linear accelerators of the same vendor and series, because of inherent problems in manufacturing and assembly, and the complexity of the components.^(1)^ Additionally, they can be altered during installation and beam tuning. After the installation of an accelerator and TPS in a clinic/hospital, therefore, acceptance testing and commissioning of the systems are required to validate the beam data. The beam parameters of the TPS are usually modeled through data from Monte Carlo simulation or measurement. Generally the beam modeling and commissioning require both scanned and nonscanned data.^1^, ^6^ For the scanning data, a water tank with a three‐dimensional scanning system is often used. The measurement should be appropriately performed to achieve accurate dose calculation. Currently several commercial scanning systems are available. These systems are required to provide identical data under the same conditions for a machine. However, there is no report that has compared the differences among various scanning systems, as institutions do not acquire more than one scanning system due to the high cost, infrequent usage, and storage issues. The quality assurance (QA) of scanning systems for beam data have not been compared or published; however, such process for scanning densitometer has been elaborated by Holmes and McCullough.^(7)^ Fortunately there was a window of opportunity to compare four different major water phantom scanning systems during a scanning workshop that enabled us to perform this study. In this report, we demonstrate the intra‐ and intervariability of beam data measured with four commercial scanning systems.

## II. MATERIALS AND METHODS

### A. MTMI educational workshop

During an educational and training workshop conducted in 2012 by the Medical Technology Management Institute (MTMI; Milwaukee, WI) at Mary Bird Perkins Cancer Center (Baton Rouge, LA), four vendors that have scanning water phantom were invited: IBA Dosimetry GmbH (Schwarzenbruck, Germany), PTW (Freiburg, Germany), Standard Imaging (Middleton, WI), and Sun Nuclear Corporation (Melbourne, FL). These vendors participated by bringing their own water tank, software, and ion chambers to demonstrate the operation and data collection process. Details of tank design and characteristics can be acquired from respective websites. A few features are summarized in Table 1 for these scanning systems. There are four linear accelerators at the center where the workshop took place. Each vendor was assigned a linear accelerator room along with a senior physicist who had expertise and working knowledge of the system operation. Scanning systems were set up and operated according to the vendor protocols with the help from a representative from each vendor. Setup, operation, software familiarization, and accuracy in data collection were the primary goals of the training session. At the end of the session, the beam data of one linear accelerator were measured with each system to evaluate intervariability in collected data.

**Table 1:** Details of the water tanks and chambers used for measurements

	*IBA*	*PTW*	*SI*	*SN*
Water Tank				
Name	Blue Phantom	MP3	DoseView 3D	3D Scanner
Shape	cubic	cubic	cubic	cylindrical
Scan size (cm)	48×48×41	60×50×40.8	50×50×41	65 dia×40
Tank setup	manual	manual	manual	automatic
$40\times 40\;{\text{cm}}^{2}$ scanning	shift/software	shift/software	shift/software	shift/software
Detector for soft‐wedge	LDA‐99	LA‐48	NA	water proof profiler
Chamber Used				
Type	CC13	TN 31010	Exradin A18	CC13
Sensitive volume	$0.13\;{\text{cm}}^{3}$	$0.125\;{\text{cm}}^{3}$	$0.123\;{\text{cm}}^{3}$	$0.13\;{\text{cm}}^{3}$

SI=Standard Imaging; SN=Sun Nuclear; dia=diameter; NA=not applicable.

### B. Measurement and assessment

During the training sessions, vendors were asked to measure chamber polarity effects, and the intravariability in percent depth dose (PDD) and profiles as a demonstration of TG‐106.^(1)^ Data were requested from each vendor and analyzed for accuracy without manipulations. Dose profiles were measured with Blue Phantom^2^ (IBA Dosimetry), MP3 (PTW), DoseView (Standard Imaging), and 3D SCANNER (Sun Nuclear) systems. A CC13 ionization chamber (IBA Dosimetry), Semiflex ionization chamber Model 31010 (PTW), or Exradin A18 chamber (Standard Imaging) was used for measurement. Each water tank was positioned with the source‐to‐surface distance (SSD) of 100 cm.

To assess the intervariability, PDD and off‐center ratio (OCR) of one accelerator were measured with each scanning system sequentially. The text file data exported from software that operate scanning phantoms were imported into a software developed in‐house with Microsoft Visual C++. To calculate the dose difference, values of secondary profiles at the identical depth or distance to the primary profile were calculated using linear interpolation. The one‐dimensional distance to agreement (DTA),^(8)^ which is the smallest distance between a measurement point and a point in the reference data with the same absorbed dose, was also calculated with the linear interpolation. The repeated measurements of PDD for intravariability assessment were conducted at other institutions. The PDDs on one linear accelerator with 6 MV X‐ray were measured several times (three to four), and the standard deviation (SD) of the values were calculated. To evaluate the polarity effect of ionization chambers, PDDs were measured with positive and negative bias of the scanning system electrometer.

## III. RESULTS

**Figure 1:**
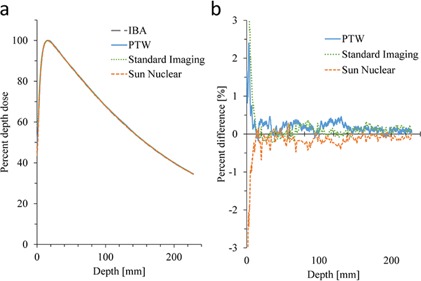
Percent depth dose (PDD) (a) of one treatment unit measured with various scanning systems. The difference (b) of the PDD values compared to the data measured with IBA scanning system chosen arbitrary as a reference.

Figure 1(a) shows the PDD for 6 MV X‐ray with $10\times 10\;{\text{cm}}^{2}$ field size measured with various scanning systems. Figure 1(b) shows the percent difference between the PDD of an arbitrarily selected system (IBA) to other scanning systems. The mean± SD of differences in the buildup region were 0.69%±0.70% (range −0.11%–+2.40%), −1.27%±1.46% (range −5.99%–+0.10%), and 2.60%±3.55% (range −0.04%–+14.6%) for PTW, Sun Nuclear, and Standard Imaging phantoms, respectively. In contrast, small differences were observed at a region deeper than maximum depth, $d_{\text{max}}$ (1.5 cm). The differences were 0.16%±0.11% (range −0.18%–+0.46%), −0.13%±0.12% (range −0.68%–+0.29%), and 0.08%±0.12% (range −0.22%–+0.37%) for PTW, Sun Nuclear, and Standard Imaging systems, respectively.

**Figure 2:**
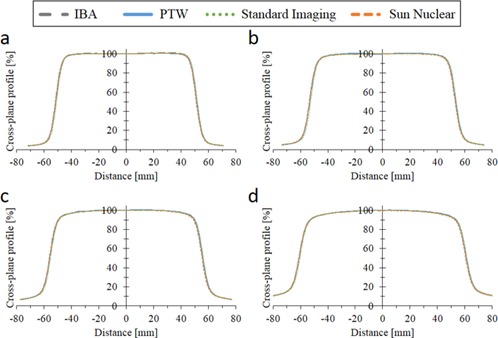
Off‐center ratio (OCR) of the same treatment unit measured with various scanning systems at (a) 1.5 cm, (b) 5.0 cm, (c) 10.0 cm, and (d) 20.0 cm depth.

Figure 2 shows the OCR at 1.5, 5.0, 10.0, and 20.0 cm depth measured with various scanning systems. All systems showed almost identical profiles, although slight displacements were observed probably due to the phantom setup. The dose differences within 80% (±40% from central axis) of radiation field between IBA and other systems are listed in Table 2. The average and maximum differences were within ±0.35% and 0.96%, respectively. The dose differences in the tail region (120%–130% of half radiation field from central axis) are listed in Table 3. Small differences (within ±0.77%) among the scanning systems were observed.

**Figure 3:**
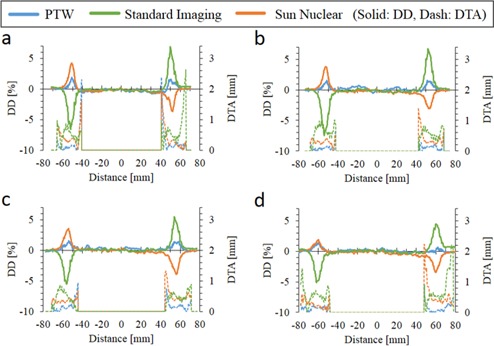
Dose difference (DD) and distance to agreement (DTA) of the off‐center ratio (OCR). The IBA scanning system was chosen arbitrarily as a reference system for comparing data. DTA data are illustrated at the region between 80%–120% of the half radiation field from central axis.

To evaluate the penumbrae region, DTA was analyzed at the region between 80%–120% of the half field from central axis. Figure 3 shows the dose difference (DD) and DTA of the OCR between the data of arbitrarily selected system (IBA) and those of other systems. The DTA of the region where the DD exceed ±1% was less than 1 mm. Therefore, all systems showed identical profiles within 1% of dose difference or 1 mm of DTA.

**Table 2:** Percent difference of the off‐axis ratio between IBA and other systems inside the 80% of radiation field. Mean± SD (range) inside the 80% of radiation field are shown

*Depth (mm)*	*PTW*	*Standard Imaging*	*Sun Nuclear*
15	−0.35±0.21(−0.80–+0.05)	−0.21±0.14(−0.54–+0.03)	−0.30±0.18(−0.72–+0.15)
50	0.12±0.27(−0.50–+0.68)	−0.17±0.15(−0.54–+0.22)	−0.29±0.19(−0.70–+0.15)
100	0.19±0.19(−0.10–+0.83)	0.02±0.13(−0.26–+0.34)	−0.09±0.19(−0.45–+0.34)
200	0.05±0.18(−0.36–+0.55)	−0.09±0.19(−0.45–+0.34)	−0.27±0.26(−0.96–+0.18)

**Table 3:** Percent difference of the off‐axis ratio between IBA and other systems inside the 120%–130% area of half radiation field. Mean± SD (range) inside the 120%–130% area of half radiation field in both positive and negative directions

*Depth (mm)*	*PTW*	*Standard Imaging*	*Sun Nuclear*
15	−0.03±0.03(−0.07–+0.03)	0.24±0.15(−0.08–+0.41)	0.09±0.10(−0.08–+0.26)
50	−0.03±0.04(−0.13–+0.03)	0.06±0.19(−0.36–+0.29)	−0.01±0.15(−0.25–+0.17)
100	−0.04±0.04(−0.12–+0.01)	0.11±0.19(−0.30–+0.35)	−0.04±0.18(−0.30–+0.17)
200	0.04±0.09(−0.17–+0.09)	0.46±0.23(+0.04−+0.77)	0.01±0.19(−0.31–+0.25)

**Figure 4:**
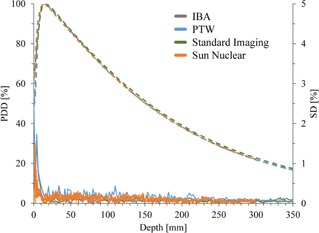
Reproducibility of percent depth dose (PDD) as measure of intrasystem variability measured with various scanning systems. Solid and dash lines represent standard deviation (SD) of three or four measurements and representative PDD profiles, respectively.

Figure 4 shows SD of PDD profiles measured three or four times for intercomparison. Representative one PDD curve is also shown for each scanning system. Because these data were not acquired for identical treatment units, slight differences are observed in PDD curves. Although large SD was observed at buildup region, the SD at the other region was less than 0.5%.

**Figure 5:**
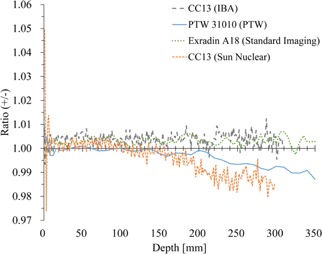
The ratio of the percent depth dose measured with positive and negative bias for electrometer is illustrated as the polarity effects.

In Fig. 5, the polarity effects were plotted with the depth very similar to the data presented in TG‐106.^(1)^ The differences among these chambers were within 1% at shallow region ≤19 cm, except buildup region. Although the Sun Nuclear and IBA scanning systems used the same type of ionization chambers, differences ≥1% were observed at deeper depths.

## IV. DISCUSSION

In current study, we compared dose profiles of one linear accelerator measured with four commercial scanning systems for intervariability in commissioning beam data. As shown in Figs. 1 to 3, every system provided very similar beam data, indicating the constancy in measurements thus a confidence in commissioning beam data of a linear accelerator. We also evaluated the intravariability with repeated measurement of PDD. The SDs of the values were smaller than 0.5% outside of the buildup region. This is interesting, as such data do not exist in literature, and it provides a limit of our accuracy by a system. Most scanning systems provide accurate data, even though vendors were not requested to use certain protocols, such as step size, data collection time, scanning method (continuous or step‐by‐step measurement), scanning order, type of ion chamber, or electrometer bias to chamber.

Some empirical‐ or correction‐based algorithms are based on the measured PDD and OCR profiles. Precise measurement of dose profiles is essential for accurate modeling of beam data in TPS. Commissioning of TPS is also essential to conduct safe and accurate radiation treatment. Several recommendations for quality assurance of TPS have been published by various national and international organizations.^1^, ^6^, ^9^, ^10^, ^11^ In 2004, European Society for Therapeutic Radiotherapy and Oncology (ESTRO) published Booklet No. 7^(6)^ as a more practical recommendation for commissioning of TPS. In 2008, the Report of the Task Group 106 (TG‐106) of the Therapy Physics Committee of the American Association of Physicists in Medicine (AAPM)^(1)^ was published that reviewed the practical aspects, as well as the physics, of linear accelerator commissioning. The report provides a confidence limit in our data collection for beam commissioning with proper tools without significant errors due to individual knowledge. We demonstrated that the differences between each scanning system were negligibly small, indicating that all systems could provide reliable and identical data irrespective of scanning system.

Several studies have compared the polarity effect of various ionization chambers.^1^, ^12^, ^13^, ^14^ In the present study, the same type of chambers (CC13, IBA Dosimetry) was used for measurement with IBA and Sun Nuclear scanning systems. These chambers showed slight different polarity effects, although the differences were small at shallow region ≤19 cm. As recommended in TG‐106, users should confirm the polarity effects of the ionization chamber before measurement.

There are many other aspects of scanning tank, such as limit on field size, star pattern, soft wedge, electron beam, point dose, and absolute dose measurements, that were beyond the scope of this study. However, some thoughts are provided here. Large field ($40\times 40\;{\text{cm}}^{2}$) scanning at 40 cm depth with over scan factor of 5 cm might limit some scanning systems (Table 1), but most of them have work‐around to collect accurate data. For large fields when off‐setting tank, limited scatter side is often a concern. However, the data provided by Srivastava et al.^(15)^ showed that the magnitude of side scatter is <1% for at least 5 cm side scattering medium. Similarly, star pattern measurements needed in some TPS may require tank manipulation, which was beyond the scope of this manuscript.^(1)^ Small field dosimetry is challenging where each vendor provides suitable detector and technique to collect data.^(16)^ The placement of reference detector in small field may be problematic; however, simply placing reference detector at the bottom of tank in the water, away from shadow of arm and detector, could be a good practice along with other innovative methods, such as time integration method. Measurements of soft‐wedges require additional detectors that were not evaluated in this study. For electron beam measurements, if an ion chamber is used, each vendor provides software to convert ionization to dose. Additionally, electron diodes could be used to avoid conversion errors.

## V. CONCLUSIONS

It is concluded that four major water phantom scanning systems provide adequate accuracy for beam data collection within 1% of dose difference or 1 mm of DTA to each other. It should be noted that this error includes uncertainties due to the phantom setup and the difference of the protocol, such as step size, measurement time, and scanning methods. The selection of device should be based on institutional comfort and personal preference of software and hardware, as well as financial considerations.

## ACKNOWLEDGMENTS

We would like to thank to Dr. J. Ed Barnes of Medical Technology Management Institute (MTMI) and scanning system vendors (IBA Dosimetry, PTW, Standard Imaging, and Sun Nuclear) for providing scanning data collected at MTMI training workshop. We are also thankful to Walter Tang for providing additional data.

## Supporting information

Supplementary Material
