# Supplementary material for: Intra‐ and intervariability in beam data commissioning among water phantom scanning systems
Source: J Appl Clin Med Phys. 2014 Jul 8;15(4):251–8. doi: 10.1120/jacmp.v15i4.4850 (PMC5875503; doi:10.1120/jacmp.v15i4.4850)
Supplement: Supplementary file 1 — Supplementary Material [file ACM2-15-251-s001.doc]

**Intra- and inter-variability in beam data commissioning among water phantom scanning systems**

Yuichi Akino,^1^ John P. Gibbons,^2^ Daniel W. Neck,^2^ Connel Chu,^2^ Indra J. Das^1^

*^1^Department of Radiation Oncology, Indiana University School of Medicine, Indianapolis, Indiana 46202.*

*^2^Department of Medical Physics, Mary Bird Perkins Cancer Center, Baton Rouge, LA 70809.*

[yakino@iupui.edu](mailto:yakino@iupui.edu) (Yuichi Akino, corresponding author)

[jpgibbons@marybird.com](mailto:jpgibbons@marybird.com) (John P. Gibbons)

[danieln@marybird.com](mailto:danieln@marybird.com) (Daniel W. Neck)

[idas@iupui.edu](mailto:idas@iupui.edu) (Indra J. Das)

**Running title:** Variability among scanning water phantoms
